# Mapping the task-general and task-specific neural correlates of speech production: Meta-analysis and fMRI direct comparisons of category fluency and picture naming

**DOI:** 10.1162/IMAG.a.154

**Published:** 2025-09-26

**Authors:** Gina F. Humphreys, Matthew A. Lambon Ralph

**Affiliations:** MRC Cognition & Brain Sciences Unit, University of Cambridge, Cambridge, United Kingdom

**Keywords:** speech production, category fluency, picture naming, fMRI, meta-analysis

## Abstract

Improving our understanding of the neural network engaged by different forms of speech production is a crucial step for both cognitive and clinical neuroscience. We achieved this aim by exploring two of the most commonly utilised speech production paradigms in research and the clinic, which have been rarely, if ever, compared directly: picture naming and category fluency. This goal was achieved in this two study investigation through a full ALE meta-analysis as well as a targeted fMRI study. Harnessing the similarities and differences between the two tasks offers a powerful methodology to delineate the core systems recruited for speech production, as well as revealing task-specific processes. The results showed that both tasks engaged a bilateral fronto-temporal speech production network, including executive and motor frontal areas, as well as semantic representational regions in the ATL, bilaterally. In addition, it was found that the extent of relative frontal lateralisation was task-dependent with the more executively-demanding category fluency task showing augmented left hemisphere activation. The results have implications for neurocomputational speech production models and the clinical assessment of speech production impairments.

## 1. Introduction

Speech production is phenomenal human capacity. Yet, despite decades of neuropsychological and imaging research it is still unclear how the brain transforms a non-verbal thought into speech output. We know that semantically driven speech production involves multiple regions; however, what we do not understand fully is the contribution of each of the key areas and pathways to the process. This is important for both theory and clinical assessment. Here, we directly investigated this issue using two of the most commonly utilized speech production paradigms in research and the clinic: picture naming and category fluency. These two tasks are ideal, firstly because they are commonly used and influential to neurocognitive theory and in clinical practice. Furthermore, the tasks are well-matched in terms of speech production (both involve single word meaningful speech production) making it a particularly helpful comparison. Specifically, the two tasks engage some common core-speech production components (e.g., semantics, phonology, etc.), but differ fundamentally in how the task is achieved: picture naming is driven bottom-up by external visual input, whereas category fluency requires top-down executive and working memory systems to guide self-generated semantic targets. Indeed, harnessing the similarities and differences between tasks offers a powerful methodology (Friston & Price, 2011) to reveal the neural correlates of the core language and cognitive systems recruited for speech production, as well as the task-specific processes.

This work has novelty and importance for three major reasons. 1) Implications for neurocognitive theory: from a basic science perspective, picture naming and category fluency are commonly used to examine the neural systems engaged by production, and have had a major influence on neurocognitive models of speech production (e.g., Indefrey, 2011; Indefrey & Levelt, 2004). 2) Clinical implications: from a clinical perspective, one or both tasks are frequently used as a proxy for speech production and/or executive function as part of neuropsychological assessment for patients with acute or neurodegenerative disease, as well as to guide resection during awake neurosurgery (e.g. Rofes et al., 2017; Sierpowska et al., 2022). It is, therefore, of clinical importance to understand their precise neural bases. 3) Methodological reasons: whilst category fluency and picture naming are frequently used tasks in fMRI (indeed, we identified 106 imaging studies for the current meta-analysis with the majority using fMRI), from a methodological perspective, existing neuroimaging paradigms are frequently suboptimal at detecting whole-brain effects, therefore potentially missing key production regions. Achieving full-brain coverage is important not only for developing fully specified neuroanatomical models but especially if one is to use fMRI as a neurosurgical tool for pre-operative planning. In the remainder of the Introduction, we will discuss each of these three key motivations in turn.

### 1.1 What are the implications of the current research for neurocognitive theory?

Geschwind’s classical model (1965) emphasized the importance of a left-lateralised dorsal network for speech production, involving posterior temporo-parietal cortex (mainly angular gyrus (AG)) and inferior frontal gyrus (IFG) connected via the arcuate fasciculus. Although Geschwind was inspired by Wernicke, recent revisits of Wernicke’s work indicate that Wernicke actually emphasized the important and, perhaps, dominance of the ventral route (Weiller et al., 2011). Contemporary theories tend to follow a “dual-stream” approach, with the majority of models emphasizing the importance of the dorsal route for speech production, whereas the ventral temporal pathway is proposed mainly for receptive speech comprehension. Specifically, in the dorsal route, lexical-semantic and phonological access is achieved via the posterior temporal-parietal cortex, after which information is transferred to the IFG via the arcuate fasciculus wherein phonological information is transformed into articulatory code for motor output (Friederici, 2002; Hagoort, 2005; Hickok, 2012; Hickok & Poeppel, 2007; Hickok et al., 2011; Indefrey & Levelt, 2004). This approach has had a broad and influential impact on the literature, yet despite the dominance of the dual-stream approach, such models potentially ignore the importance of certain key structures, such as the anterior temporal lobe (ATL), and potentially mischaracterises the role of other areas, including the AG and the lateral frontal cortex. We will discuss each of these in turn below.

#### 1.1.1 The role of the anterior temporal lobe (ATL) in speech production

In the dual-stream approach, ventral areas, such as the ATL, often have no/minimal proposed involvement in production (Hickok, 2012; Hickok & Poeppel, 2007). This situation is heavily influenced by neuroimaging findings; for instance a large review highlighted reliable fronto-posterior temporal activation for speech production tasks using PET/fMRI or EEG/MEG (Indefrey, 2011; Indefrey & Levelt, 2004). The patient literature, however, paints a very different and clear picture. Here, damage to the ventral pathway, specifically to the ATL in semantic dementia (SD) or temporal lobe resection, results in a pronounced deficit in meaningful speech production, comprehension, as well as non-verbal semantic tasks (Lambon Ralph, et al., 2012; Lambon Ralph et al., 2017). This is in keeping with the proposal that the ATLs act as a semantic hub that supports transmodal conceptual representation, and is critical to speech production (Jefferies & Lambon Ralph, 2006; Lambon Ralph et al., 2001, 2012, 2017). Indeed, this theory has gained converging support across a variety of methodologies, including TMS, fMRI, ECoG, as well as from computational modeling (Lambon Ralph et al., 2017; Lambon Ralph, Sage, et al., 2010; Pobric et al., 2010; Rogers et al., 2021; Sato et al., 2021; Shimotake et al., 2014; Woollams et al., 2017). Alternative dual-route language frameworks that incorporate an ATL-semantic pathway, implemented in computational models (Ueno et al., 2011), demonstrate key divisions of labour across the dorsal and ventral pathways, with the ventral crucial for semantically-driven speech production and the dorsal for repetition and extraction of the phonological structures of the language. If true, one should expect the ATL to be engaged by both picture naming and category fluency tasks.

#### 1.1.2 The role of the angular gyrus (AG) in speech production

It has been proposed that the AG acts as a multi-modal semantic storage hub, similar in function to the ATL (Binder et al., 2009; Geschwind, 1965), based largely on neuroimaging evidence showing that the AG reliably shows relative differences in levels of deactivation in favour of word>non-words or concrete>abstract (Binder & Desai, 2011; Binder et al., 2009; Humphreys & Lambon Ralph, 2017). Nevertheless, the majority of evidence comes from receptive rather than expressive language tasks. Those tasks that have included speech production, have not consistently found AG engagement and recent work suggest that the minority of positive production tasks might relate to autobiographical recall (Geranmayeh et al., 2012; Humphreys et al., 2024). If the AG is a core component of the speech production system, then it should be activated by both the category fluency and picture naming tasks.

#### 1.1.3 The role of the lateral frontal cortex in speech production

In the standard dual-stream framework, the lateral frontal cortex is primarily considered to be involved in articulatory motor planning (Hickok, 2012; Hickok et al., 2011). Yet the lateral frontal cortex is a heterogeneous region packed with multiple additional functions related to speech production, such as phonological and executive processes. In terms of executive mechanisms, sections of the dorsal lateral prefrontal cortex are involved in domain-general executive control and form a key part of the multiple demand (MD) network (Fedorenko et al., 2013), and the IFG, specifically within *pars triangularis* (BA45), forms part of the semantic control network, a network involved in the use and manipulation of semantic information (Jefferies & Lambon Ralph, 2006; Lambon Ralph et al., 2017). Indeed, damage to the IFG can impair performance in executively demanding expressive and receptive semantic tasks (Jefferies & Lambon Ralph, 2006; Noonan et al., 2010; Rogers et al., 2015), and similar results have been found using TMS in healthy participants (Krieger-Redwood & Jefferies, 2014; Whitney et al., 2010). Likewise, recent work with continuous speech production has shown a key role for ventral IFG in semantically coherent speech production (Hoffman, 2019; Hoffman et al., 2018). In terms of current study, category-fluency places multiple top-down demands on the executive systems, including linguistic and non-linguistic regions involving working memory, search mechanisms, and the inhibition of inappropriate or already produced items. In contrast, picture naming is driven by bottom-up visual input; therefore, in addition to core-production regions, it relies largely on the visual object recognition system with relatively lower executive demands. We, therefore, expect that category fluency will more strongly/widely recruit frontal executive systems.

### 1.2 What are the clinical implications of the current research?

From a clinical perspective, category-fluency and/or picture naming tasks are commonly used tools to assess speech production and executive functions in many different types of patient groups including acute (e.g., stroke), neurodegenerative or neurosurgical (e.g., glioma or temporal lobe sclerosis). Indeed, a general survey of 21 neurosurgical centers across 11 European countries found that fluency and naming were the most frequently used tests to assess language performance pre-surgically and also during awake craniotomies to map language function (Rofes et al., 2017). Their ease of use makes them a practical clinical tool to assess speech and language deficits at the bedside post-stroke, or for use in the neurological clinic to aid in the diagnosis of neurodegenerative disorders, and to guide neurosurgical mapping before and/or during neurosurgical resection (Sierpowska et al., 2022). In a neurosurgical setting, understanding the cognitive and language role of each region has immediate implications for whether the region is included in the resection or the neurosurgical approach taken to access deeper tissue. Accordingly, understanding the neurocognitive make-up of these clinical tasks is key to effective clinical assessment both pre- and during surgery, and the subsequent likely neurosurgical effects on cognitive function. For example, following the demonstrated importance of the ventral ATL in language functions (the basal temporal language area: (Lüders et al., 1991)) enhanced understanding and mapping of the ventral ATL to semantic function has led to changes in neurosurgical practices, resulting in better language outcomes for the patients (Mikuni et al., 2006).

#### 1.2.1 Production laterality

Clinically, language production is frequently considered a largely left hemisphere function (Eggert, 1977; Geschwind, 1972; Lichtheim, 1885). Nevertheless, with advances in functional imaging there is increasing evidence of a bilateral albeit asymmetric pattern of activation in speech production (Blank et al., 2002; Geranmayeh et al., 2012; Humphreys et al., 2024; Moore & Price, 1999; Zelkowicz et al., 1998). To further muddy the waters, the extent of left vs. bilateral activation appears task-dependent (Birn et al., 2010; Blank et al., 2002; Geranmayeh et al., 2012; Moore & Price, 1999; Vitali et al., 2005; Zelkowicz et al., 1998). The extent to which speech production lateralizes has implications for patient treatment, such as whether or not to assess language function during neurosurgery and/or not to refer a patient to speech and language therapy. For instance, in a neurosurgical setting, patients undergoing right-hemisphere surgery tend to have larger volumes of tissue resected compared to left-hemisphere cases (Rice et al., 2018). Therefore, using two very different tasks we are able to help to determine the extent to which language laterality might vary depending on the task.

#### 1.2.2 Speech assessment in patients

Picture-naming and category fluency are very practical in the clinic since they are quick to perform and require relatively little technology, and thus make an attractive task for language screening (Henderson et al., 2024; Sierpowska et al., 2022). Issues may arise, however, if only one task is used in isolation as a generalized proxy for speech production, as patients can show varying performance across measures, for instance some patients may be severely impaired in category fluency assessments compared to picture naming due its enhanced executive demands rather than to language impairment per se (Noonan et al., 2010; Robinson et al., 1998; Satoer et al., 2022; Satoer, Visch-Brink, et al., 2014; Satoer, Vork, et al., 2014; Schumacher et al., 2022). Indeed, given its multiple cognitive demands, it is entirely possible to have poor category and letter fluency scores as a result of entirely non-language impairments (Henderson et al., 2024; Hodges & Patterson, 1995). In the neurosurgical setting, one would seek to preserve as much function as possible; however, these neurosurgical clinical inferences are dependent on the nature of task. For instance, an over-reliance on picture-naming alone could downplay the relevance of executive functions that are, nevertheless, critically important for language. Indeed, pre- and post-operative picture naming performance has been shown to be normal in many glioma patients (Satoer, Visch-Brink, et al., 2014; Satoer, Vork, et al., 2014), but category fluency shows long-term impairment (Satoer et al., 2022). Alternatively, an overreliance on category fluency might downplay the role of the right-hemisphere and/or the visual system. Therefore, by simultaneously mapping the similarities and differences in the networks engaged by each task, the current study should provide helpful information for clinical decisions.

### 1.3 Current methodological issues in the literature

Our ability to derive accurate neurocognitive theory and clinical-mapping of the speech production system relies on the ability to reliably and maximally activate the key neurocognitive system. Despite a number of neuroimaging studies investigating speech production, there are a number of key methodological limitations in the current literature. First, as mentioned above, the ATL is highly susceptible to signal dropout using conventional fMRI paradigms (Halai et al., 2014) and is much more likely to be observed when contrasted against an active task baseline than rest (Humphreys et al., 2015; Visser et al., 2010). These and other factors mean that the current fMRI speech production literature may well be missing the ATL from the full production network. Indeed, ATL activation has been observed during picture naming tasks using PET (Blank et al., 2002; Moore & Price, 1999; Zelkowicz et al., 1998), a technique that does not suffer from signal dropout. Achieving full-brain coverage is important not only for developing neuroanatomical models but especially if one is to use fMRI with patients to assess the functional integrity of the ventral language pathways (e.g., in aphasia or as a neurosurgical tool for pre-operative planning: (Rice et al., 2018; Robson et al., 2014))especially as the ATL is commonly resected in the neurosurgery for temporal lobe epilepsy and gliomas tend to be found more commonly in frontotemporal regions. Secondly, existing imaging paradigms often use covert rather than overt speech production which will limit the extent of the observed the production network. Thirdly, existing studies tend to suffer from a lack of experimental control. For instance, category fluency studies often use very long blocks and/or do not take into account the speech rate or the number of words produced (e.g., Amunts et al., 2004; Gaillard et al., 2003; Gurd et al., 2002; Kircher et al., 2009; Marsolais et al., 2015; Paulesu et al., 1997; Vitali et al., 2005). In order to make a direct comparison one has to consider how to make the tasks as comparable as possible in terms of influential factors such as speech rate, number of words produced, etc.

#### 1.3.1 Aims

Here, we conducted the first direct comparison of picture naming and category fluency tasks via two major, linked investigations: (1) a meta-analysis of the existing picture naming and category fluency literature, as well as (2) the first within-subject fMRI study to compare the tasks directly. Meta-analyses are powerful techniques to identify reliable findings from the existing literature that hold across investigations and are independent of study-specific factors and other idiosyncrasies. By their very nature of relying on the existing literature, however, meta-analytic results can be biased by any methodological and sampling issues that systematically occur across studies (of which there are several in this case). Hence, combining the advantages of meta-analyses with those from a well-controlled within-subject fMRI study is the optimal approach. In our fMRI study, we controlled for these methodological issues by: 1) using a dual-echo fMRI protocol which improves ATL signal detection (Halai et al., 2014); 2) using overt speech production tasks; and 3) matching the rate and the number of words produced across tasks by using a paced paradigm.

The current approach allows: 1) the use of two very different speech production methodologies allows greater precision when determining the shared cognitive mechanism and associated brain regions across tasks using conjunction analyses (e.g., semantic, phonological, and motor components), and also to delineate task-specific processes through direct task contrasts; 2) we assessed the extent to which the production network is left lateralized (vs. bilateral), and whether the extent of lateralisation is task-dependent in the whole-brain and targeted ROI analyses; and 3) to assess the “semantic hub hypothesis” for the ATL and AG: if these areas are critical for semantic retrieval, then one would expect activation in both fluency and naming tasks.

## 2 Methods

### 2.1 Meta-analysis

#### 2.1.1 Study selection

We conducted a highly extensive search of the existing imaging literature in order to be as inclusive as possible. The picture naming and category-fluency studies included in the meta-analysis were identified by keyword searches Google Scholar (these included “Speech production” or “Word production” or “Naming’ or “Fluency” AND “fMRI” or “PET”), this was supplemented by a search of studies that have been uploaded to https://neurosynth.org/studies/ using the search terms “production”, “fluency”, or “naming”. Together, this resulted in the identification of over 500 studies. Of those studies, only those using fMRI or PET that included either/both a picture naming and category-fluency task and reported peak activation in standard space (Talairach or MNI) based on whole-brain statistical comparisons were included. This resulted in the identification of 62 picture-naming studies (628 foci), and 44 category-fluency studies (407 foci).

#### 2.1.2 ALE analyses

The ALE analyses were carried out using GingerAle 3.0.2 (Eickhoff et al., 2009; Laird et al., 2005). All activation peaks were converted to MNI standard space using the built-in Talairach to MNI (SPM) GingerALE toolbox. Analyses were performed with voxel-level thresholding at a p-value of .001 and cluster-level FWE-correction with a p-value of .05 over 10,000 permutations.

#### 2.2 fMRI study

##### 2.2.1 Participants

Twenty participants took part in the fMRI study (average age = 24.30, SD = 3.96; N female = 12). All participants were native English speakers with no history of neurological or psychiatric disorders and normal or corrected-to-normal vision. Informed consent was obtained from all participants for being included in the study.

##### 2.2.2 Task design and procedures

There were three experimental tasks presented using a randomised blocked-design. At the beginning of the block, the participants were cued with a task instruction for a duration of 1.5 seconds. Four items were presented per block, each for 2 seconds separated by a 250 ms fixation cross (block length = 10.5 seconds). There were 80 items in each task (20 blocks per condition). The blocks were randomly separated with 30 rest blocks consisting of a fixation cross with duration 10.5 seconds.

###### 2.2.2.1. Picture naming task

The participants were cued to “Name the picture” at the beginning of each block. The pictures consisted of 80 black-and-white line drawings from the Snodgrass and Vanderwart (1980) picture set and consisted of a mixture of living and non-living items. Items were randomly assigned to blocks for each participant (i.e., each participant saw items in a different order in different blocks). The participants were instructed to name the picture before it left the screen.

###### 2.2.2.2 Category fluency task

At the beginning of each block, the participants were cued with a semantic category (e.g., “Name zoo animals”). During each trial, a scrambled image was presented on the screen. There were 20 categories that were presented in a varying and randomised order across participants. The participants were instructed to generate a word every time a scrambled image was presented. This provided a method of pacing the rate of speech production to match the picture naming task. The scrambled picture also acted as a low-level visual control for the pictures in the picture naming task.

###### 2.2.2.3 Control task

On each trial, the participants were cued to “Say OK” overtly when a scrambled line drawing was centrally presented. The control trials acted as a control for motor aspects of speech production as well as low level visual processing in the experimental tasks.

##### 2.2.3 Task acquisition parameters

Images were acquired using a 3T Philips Achieva scanner using a dual gradient-echo sequence, which has improved signal relative to conventional techniques, especially in areas associated with signal loss (Halai et al., 2014). 31 axial slices were collected using a TR = 2.8 seconds, TE = 12 and 35 ms, flip angle = 95°, 80 x 79 matrix, with resolution 3 x 3 mm, and slice thickness 4 mm. Across all tasks, 359 volumes were acquired in total collected in one run of 1005.2 seconds. B0 fieldmap images were also acquired to correct for image distortion.

##### 2.2.4 Task data analysis

###### 2.2.4.1 Preprocessing and general linear modelling

The dual-echo images were analyzed using SPM12, implemented in MATLAB. Preprocessing followed included B0 fieldmap-based correction to account for susceptibility-induced distortions in the functional images, using SPM12’s fieldmap toolbox. All functional images were corrected for differences in slice acquisition time (slice timing correction), followed by motion correction using a 6-parameter rigid-body transformation and co-registration to each participant’s T1-weighted anatomical image using normalized mutual information. Normalization to standard template space was achieved using DARTEL (Ashburner, 2007), warping all images to MNI space based on the MNI ICBM 152 (non-linear 6th generation) template. Normalized functional images were resampled to 3 mm^3^ resolution using 4th-degree B-spline interpolation. Spatial smoothing was applied using an 8 mm^3^ full-width at half maximum (FWHM) isotropic Gaussian kernel.

At the first level, statistical analysis was conducted using the general linear model (GLM). Data were high-pass filtered at 128 seconds to remove low-frequency drifts. Each condition for each task was modeled with a separate regressor, and event-related responses were convolved with SPM’s canonical hemodynamic response function. Time and dispersion derivatives as well as six motion parameters estimated during realignment were entered as nuisance regressors. Serial auto correlations in the timeseries were accounted for using an autoregressive AR(1) model during Classical (ReML) parameter estimation. Motion outliers were assessed by examining absolute displacement; no participants exceeded the threshold of one voxel (3 mm), consistent with prior recommendations (Johnstone et al., 2006). At the individual level, task conditions (naming and fluency) were contrasted against a control condition as well as directly compared with each other. At the second level, contrast images from individual participants were entered into one-sample t-tests and a voxel-wise height threshold of p < .001 (uncorrected) and a cluster-level family-wise error (FWE) correction at p < .05 were applied.

###### 2.2.4.2 ROI analyses

We also conducted targeted ROI analyses. The purpose of these was twofold. Firstly, we compared activation from key left-hemisphere ROIs with their right-hemisphere homologue in order to test for laterality effects across tasks. Secondly, we compared task activation within each ROI to test our predictions regarding the functional engagement of these regions. We included four 8 mm spherical lateral frontal ROIs (two left hemisphere and two right hemisphere) in order to assess the extent to which each task engaged areas associated with executive function. Specifically, we included one ROI in the left IFG, pars triangularis (BA45) (MNI coordinates: -45, 19, 19) associated with semantic control based on the coordinates from meta-analysis of studies with high- vs. low-semantic control demands (Noonan et al., 2013), and one in the left inferior frontal sulcus using the peak group coordinate from the MD network (MNI coordinates: -41, 23, 29) (Fedorenko et al., 2013), as well as their right hemisphere homologues (MNI coordinates: IFG: 45, 19, 19; IFS: 41, 23, 29). In order to assess the functional involvement of the ATL across tasks, we also included bilateral ventral ATL ROIs based on the coordinates from a semantic study (MNI coordinates: -36, -15, -30 and 36, -15, -20) (Binney et al., 2010). Finally, we included an ROI of the left AG based on the peak coordinates from meta-analyses of semantic studies (MNI coordinates: -48, -64, 34) (Humphreys & Lambon Ralph, 2015) to test the hypothesis that the left AG acts as an additional semantic hub (Binder et al., 2009).

## 3 Results

### 3.1 Meta-analysis results

**Fig. 1.**
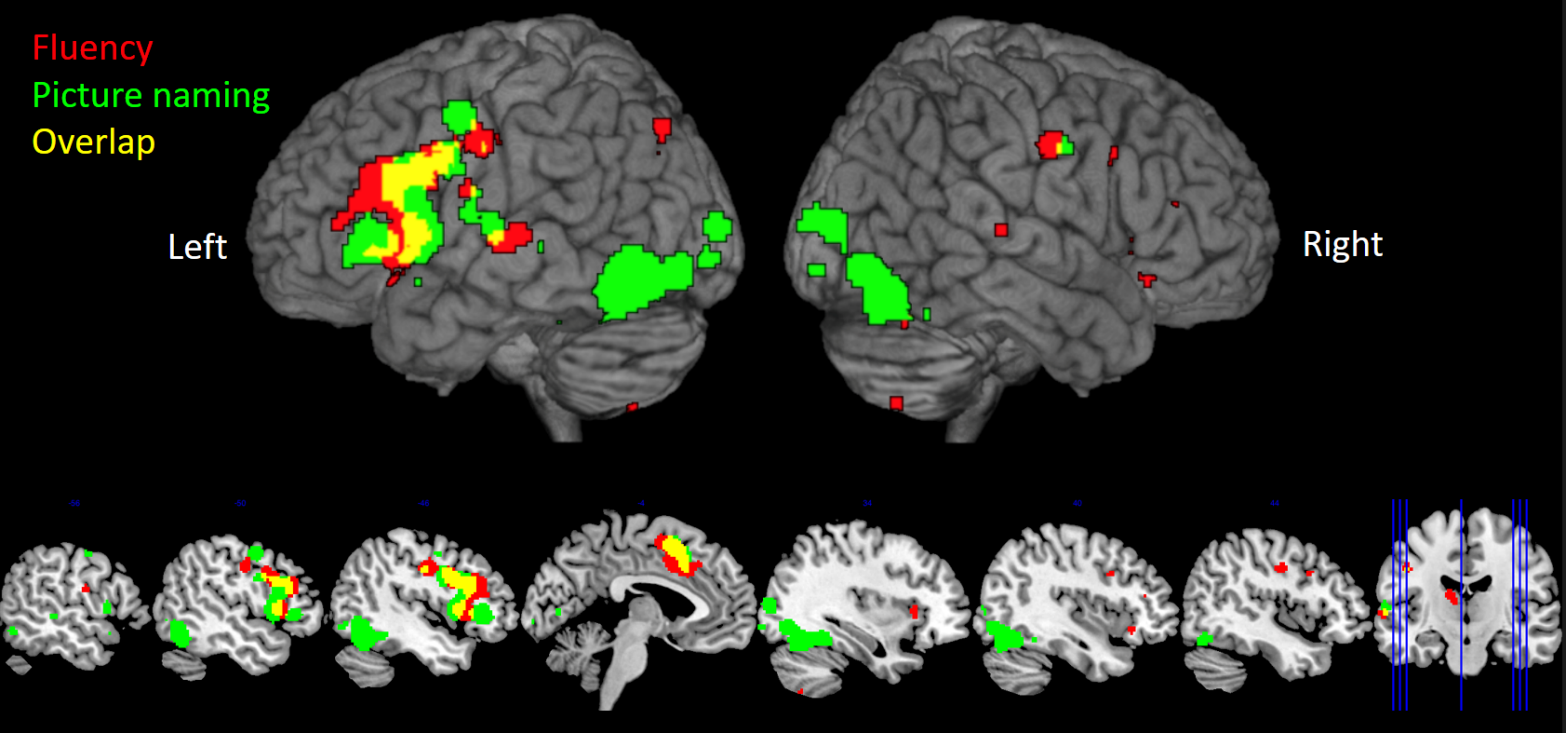
The significant clusters of activation in the meta-analysis of category fluency studies (red) and picture naming (green) (thresholded at a p < .001, cluster-level FWE-correction p < .05). The overlap is shown in yellow.

The analysis of category fluency and picture naming studies showed that both tasks reliably engaged overlapping frontal areas (inferior frontal gyrus (IFG), middle frontal gyrus (MFG), premotor and motor cortex, and the supplementary motor area (SMA)/anterior cingulate cortex (ACC)), in areas associated with executive and motor processing (Fedorenko et al., 2013; Hickok, 2012; Lambon Ralph et al., 2017). Significant right hemisphere frontal recruitment was only found for the fluency studies but not picture naming, and these clusters were comparatively smaller in comparison to those in the left hemisphere (the largest left frontal cluster was 66,144 mm^3^, compared to 3264 mm^3^ in the right hemisphere). Beyond the frontal cortex, category fluency showed very little recruitment, whereas picture naming revealed significant clusters in bilateral visual and posterior ventral temporal cortex (fusiform gyrus) in areas associated with visual object recognition (DiCarlo & Cox, 2007; Riesenhuber & Poggio, 1999) (Fig. 1, Supplementary Table S1).

A direct comparison of fluency and picture naming results showed significantly stronger recruitment of left frontal (premotor, SMA/ACC), and right frontal cortices (MFG) for the fluency compared to naming tasks, although the left hemisphere clusters were larger (the largest left frontal cluster was 4592 mm^3^, compared to 1920 mm^3^ in the right hemisphere). The direct contrast of picture naming and fluency revealed clusters in bilateral visual and posterior ventral temporal cortex (fusiform gyrus) (see Supplementary Table S1). Neither task was found to reliably activate the ATL. We propose that this may be due to there being limited fMRI signal in this area (Visser et al., 2010)—indeed, studies that used PET rather than fMRI did find ATL involvement (Blank et al., 2002; Moore & Price, 1999; Zelkowicz et al., 1998). Plus, the results of the subsequent fMRI study that used a technique for improved ATL signal detection support this conclusion (see next).

### 3.2 fMRI results

#### 3.2.1 The core production network

**Fig. 2.**
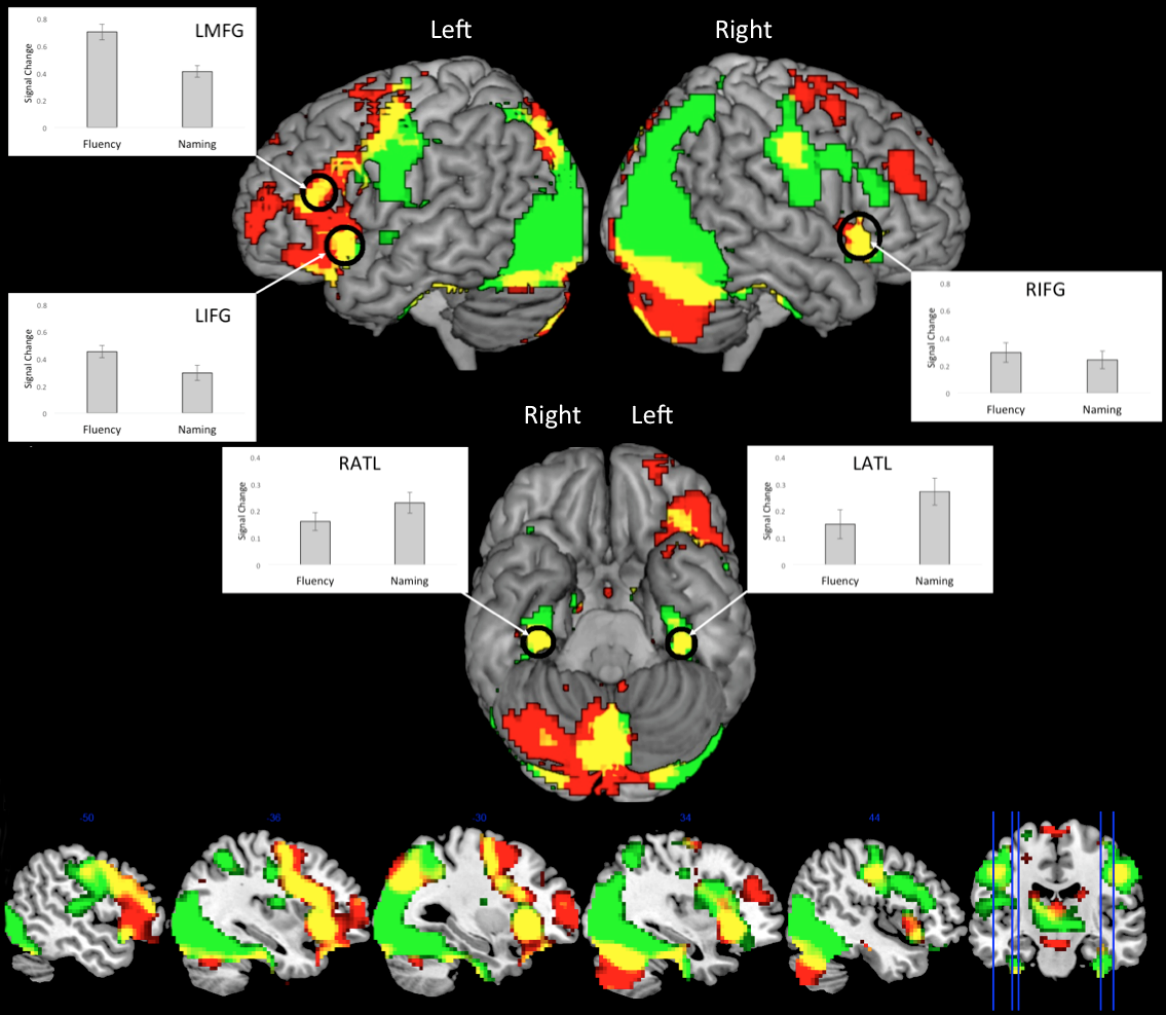
The results from the fMRI study for the contrasts Fluency > Control (red) and Naming > Control (green) (thresholded at p < .001, cluster corrected using FWE p < .05). The overlap between each network is shown in yellow. The plots show the percentage signal change relative to control for each task.

To determine a task-general network, we examined the areas of overlap between the Fluency > control and Naming > control contrasts (Fig. 2, Supplementary Table S2). A common pattern of activation was observed in bilateral frontal areas that are associated with executive and motor processes (inferior frontal gyrus, middle frontal gyrus, the supplementary motor area, anterior cingulate cortex, and pre-motor and primary-motor cortices) (Binder et al., 2009; Fedorenko et al., 2013; Hickok, 2012; Jackson, 2021). Of note, bilateral ventral ATL (anterior fusiform gyrus) was also engaged by both tasks indicating common involvement of the semantic hub across tasks (Lambon Ralph et al., 2017). In addition to this fronto-temporal network, common activation was observed in bilateral superior parietal areas previously associated with domain-general executive control and visuo-spatial processes (Fedorenko et al., 2013; Kravitz et al., 2011), as well as occipital cortex and the cerebellum. A similar network is found in the contrast of each task vs. rest (fixation), except with the addition of extensive activation of the superior posterior temporal cortex when modeling task > rest (see Supplementary Fig. S1).

#### 3.2.2 Task-varying networks

##### 3.2.2.1 Fluency > naming

**Fig. 3.**
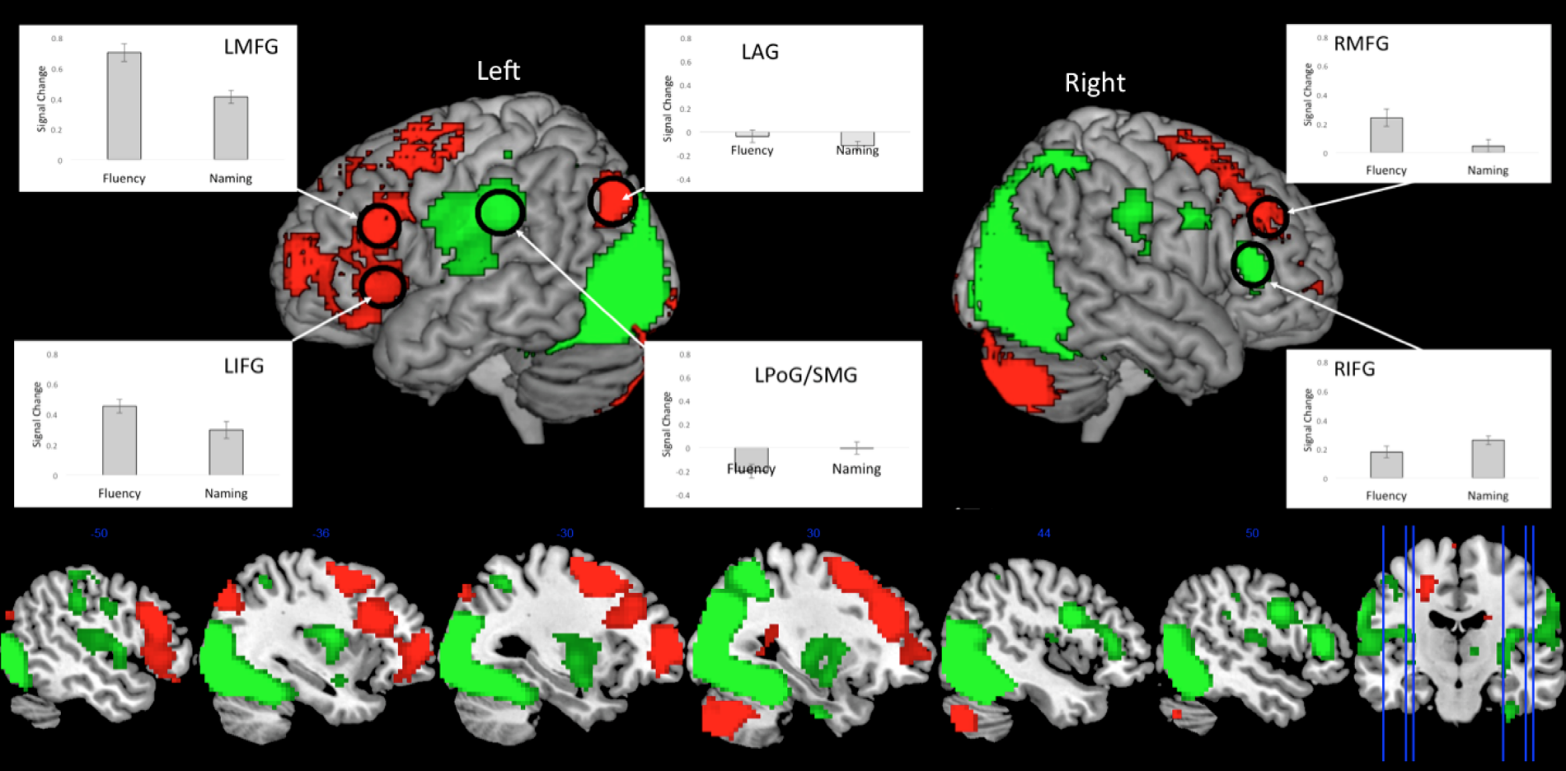
The direct contrast of Fluency > Naming (red) and Naming > Fluency (green) (thresholded at p < .001, cluster corrected using FWE p < .05). The plots show the percentage signal change relative to control for each task.

While large areas of the left frontal cortex were shown to be engaged by both fluency and picture naming, activation was found to be stronger in many areas for the fluency task compared to naming (IFG, MFG, SMA, ACC, and premotor cortex). This enhanced frontal activation is consistent with the increased executive demands of the fluency compared to naming task. This was also true in right dorso-medial frontal cortex (ACC and MFG), although these clusters were comparatively smaller compared to the left hemisphere and were restricted to dorso-lateral regions associated with domain-general executive control. While IFG is frequently associated with semantic control (Jackson, 2021), the area immediately dorsal shows a more domain-general function (Chiou et al., 2023). Outside the frontal lobe, the only region to show a fluency > naming difference was the left AG. When this AG activation is plotted, one can observe that this pattern does not, in fact, reflect *increased* activation for the Fluency task vs. the Naming tasks, rather by *enhanced* deactivation for Naming relative to the control task (whereas the Fluency task does not differ to control). The fact that the AG was either not modulated or decreased in activation suggests that this region does not play a significant role in either task, contrary to the semantic AG hub hypothesis. All results are shown in Figure 3 and Supplementary Table S2.

##### 3.2.2.2 Naming > fluency

As expected, picture naming elicited stronger activation in the bilateral occipital cortex as well as occipito-temporal activation along the length of fusiform gyrus, in areas associated with visual-object recognition (DiCarlo & Cox, 2007; Riesenhuber & Poggio, 1999). Additionally, in the frontal lobe, while the left frontal cortex was more strongly engaged by fluency, picture naming showed stronger activation for the right IFG and premotor cortex (refer to the ROI results for further investigations into the laterality differences). Unexpectedly, naming was also found to show stronger activation of bilateral postcentral/supramarginal gyrus. When activation is plotted for this region, it is shown that this difference can be explained by reduced activation relative to the control task in Fluency but no difference in Naming. Therefore, this region does not appear to play an active role in either task. All results are shown in Figure 3 and Supplementary Table S2.

### 3.3 ROI analyses

**Fig. 4.**
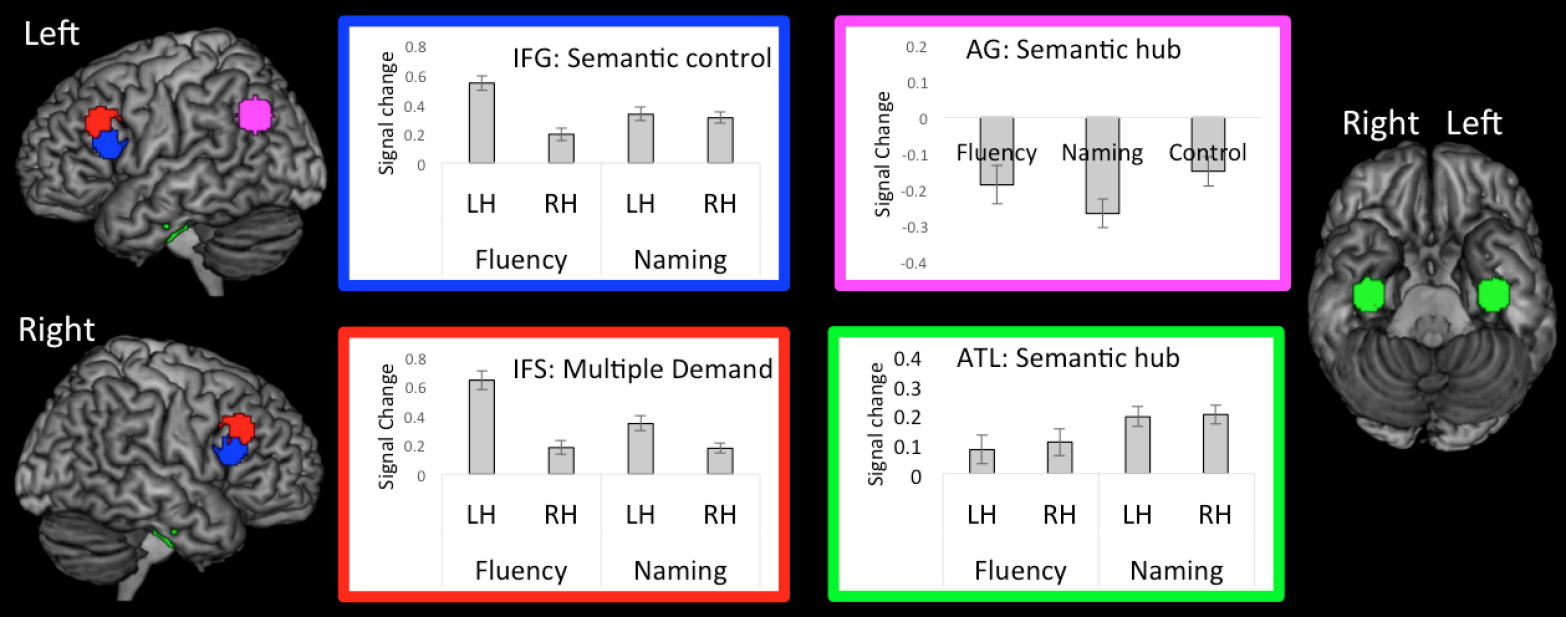
The percent signal change relative to control for each task in the IFG pars triangularis, IFS, and ATL in the left and right hemispheres, as well as each task relative to rest in the left angular gyrus (for reasons highlighted in the main text).

While language production is classically considered exclusive to the left hemisphere, the imaging literature suggests that there may be a more bilateral pattern, as well as the possibility that the extent of laterality might be task-dependent (Blank et al., 2002; Stefaniak et al., 2022; Stefaniak et al., 2020). Planned ROI analyses were conducted to examine variations in laterality across tasks in three key regions of the executive and network: including 1) the semantic hub region of the vATL, as well as two lateral frontal executive areas; 2) the semantic control region within IFG, *pars triangularis* (Jackson, 2021); and 3) the more dorsal MD control region of IFS (Fedorenko et al., 2013) (Fig. 4).

Repeated-measures ANOVAs were used to test for laterality x task effects in each ROI. The semantic control region of the IFG, *pars triangularis,* was found to show a significant effect of hemisphere (F(19) = 29.76, p < .001), no effect of task (F(19) = 2.56, p = .13), and a significant hemisphere x task interaction (F(19) = 112.05, p < .001). Pairwise comparisons showed that the interaction was explained by significantly increased left hemisphere compared to right hemisphere activation for the fluency task (t(19) = 8.77, p < .001), but no hemispheric difference for the naming task (t(19) = 1.73, p = .1), and the hemispheric difference was significantly greater for the fluency task compared to the naming task (t(19) = 10.59, p < .001). This suggests that left hemisphere activation is boosted for the fluency task whereas the naming task shows a more bilateral activation pattern. This result is consistent with outcome of the whole-brain analysis.

The MD region of the IFS showed a similar pattern as the IFG, *pars triangularis*: a significant effect of hemisphere (F(19) = 42.37, p < .001), and a significant hemisphere x task interaction (F(19) = 62.22, p < .001). But additionally showed a significant effect of task (F(19) = 15.86, p < .001). Pairwise comparisons showed that this domain-general control region was more engaged by fluency compared to naming in the left hemisphere (t(19) = 6.58, p < .001) but not in the right hemisphere (t(19) = .46, p = .91). In addition, while the left hemisphere was generally more strongly engaged than the right hemisphere by both the fluency task (t(19) = 7.91, p < .001) and the naming task (t(19) = 3.80, p < .001), the hemispheric difference was significantly greater for the fluency task compared to the naming task (t(19) = 7.89, p < .001). Again, this suggests that left hemisphere activation is particularly boosted for the fluency task, which carries greater executive demand.

For the vATL, there was significant effect of task F(19) = 8.50, p < .01), but no effect of hemisphere (F(19) = 0.28, p = .25), and no significant hemisphere x task interaction (F(19) = 0.21, p < .70). This indicated whilst there was significantly greater vATL activation overall for the naming vs. fluency task (t(19) = 2.92, p < .009), the pattern of activation was bilateral for both tasks, and is consistent with notions of the vATL operating as a bilateral semantic system (Jung & Lambon Ralph, 2016; Rice, Lambon Ralph, & Hoffman, 2015).

In addition to the hemisphere x task interaction ROI analyses, we also examined the role that the left AG played in each task relative to the control task and rest (Fig. 4). It has been proposed that the AG acts an additional semantic hub (Binder et al., 2009) and hence should be recruited by category fluency and picture-naming. The AG is also part of DMN, hence it is commonly (but not always) deactivated relative to rest. A one-way ANOVA was conducted including naming, fluency, and the control as variables and no significant effect of task was found (F(19) = 2.38, p < .11). Furthermore, unlike IFG, IFS, and ATL which exhibited task positive engagement relative to rest (all ts > 4.75, ps < .001), the AG showed task-general deactivation relative to rest (all ts > -3.57, p < .001).

## 4 Discussion

In this double-study investigation of speech production, we combined meta-analysis and fMRI to examine two of the most commonly utilized speech production paradigms in research and the clinic: picture naming and category fluency. We had three key aims. First, to use two very different speech production paradigms to determine what regions are commonly engaged across tasks, and also delineate task-specific components by directly contrasting the tasks. The results showed that both tasks engaged a shared fronto-temporal speech production network, including executive and motor frontal areas, as well as semantic representational regions in the ATL (Figs. 2 and 4). This network presumably reflects the core speech production systems. Task-specific differences were also revealed, with greater engagement of the lateral frontal executive system for the fluency task, and greater ventral occipito-temporal activation for naming. The second aim of the current study was to test the assumption that the production network is largely left lateralized (vs. bilateral), and the extent to which this is task-dependent. The results showed that both tasks engaged a bilateral fronto-temporal network; however, in lateral frontal areas, fluency was associated with boosted left hemisphere activation (Figs. 3 and 4), whereas naming showed an overall weaker but more bilateral pattern (Fig. 4). Targeted ROI analyses supported these findings. Both naming and fluency engaged bilateral lateral frontal executive regions (IFG *pars triangularis*, and IFS), although activation was stronger for the fluency in the left- relative to the right hemisphere, whereas naming showed a smaller/no hemispheric difference. The third aim of the current study was to test the hypothesis that the ATL and AG act as semantic hubs and should therefore be engaged by both fluency and naming. We found that this pattern was true of the ATL; the ATL showed bilateral engagement for fluency and naming tasks (Figs. 2 and 4) (although stronger activation for naming (Fig. 4)). In contrast, no evidence was found for the involvement of the AG in either task; in fact, both tasks showed AG deactivation relative to rest, and were either equal to or deactivated relative to the control task (Fig. 4).

### 4.1 The core speech production network

Across the two very different speech production tasks, we identified a core fronto-temporal production system. This comprised bilateral frontal cortex in regions associated with motor function and executive control (Fedorenko et al., 2013; Hickok, 2012; Lambon Ralph et al., 2017), and the bilateral ATL associated with semantic representation (Lambon Ralph et al., 2017). How do these findings compare to prominent speech production models, such as the dual-stream model (Hickok, 2012; Hickok & Poeppel, 2000, 2004, 2007; Hickok et al., 2011) and results from the patient literature?

#### 4.1.1 A bilateral speech production system

We found evidence of a bilateral yet left biased speech production system (although the degree of lateralisation is to some extent task-dependent – see below). This goes against the traditional assumption that speech production relies only on a left-lateralized system which originates from the neuropsychological literature, whereby chronic aphasia is classically associated with left- but not right-hemisphere damage (Eggert, 1977; Geschwind, 1972; Lichtheim, 1885). Indeed, a bilateral speech production network has been shown elsewhere in the neuroimaging literature (Blank et al., 2002; Geranmayeh et al., 2012; Humphreys et al., 2024; Moore & Price, 1999; Zelkowicz et al., 1998), and TMS studies using healthy participants have also shown that some right-hemisphere areas play an important role in language function (Hartwigsen et al., 2013; Jung & Lambon Ralph, 2016; Lambon Ralph et al., 2009; Pobric et al., 2010; Woollams et al., 2017). How does one reconcile these apparently contradictory findings with the patient literature? One possibility is that language production is a bilateral but asymmetric system, whereby the left-hemisphere has greater computational capacity compared to the right. Indeed, by building this principle into computational models, it has been shown that, after right-hemisphere damage, there is greater capacity within the left hemisphere to pick up the additional processing requirements, resulting in only a mild/transient language deficit. In contrast, after left-hemisphere damage, there are only the limited right-hemisphere resources available to support recovery, resulting in severe aphasia (Chang & Lambon Ralph, 2020). Consistent with this model, patients with right hemisphere damage have been found to show transient language deficits in the acute phase with milder deficits remaining chronically, implying some right-hemisphere contribution in the undamaged system (Gajardo-Vidal et al., 2018). Going beyond classical speech areas, the bilateral activation pattern for the ATLs is consistent with previous patient work, rTMS explorations, and formal computational models, implying that the ATL is even more inherently bilateral in nature, with substantial semantic deficits only after bilateral rather than unilateral lesions (Lambon Ralph, Cipolotti, et al., 2010; Lambon Ralph et al., 2012; Pobric et al., 2010; Rice et al., 2018; Schapiro et al., 2013). Indeed, existing work suggests that the left and right ATLs work together interactively and share the same core function, although some small task-related differences can arise as a consequence of differential connectivity to other regions, with the left ATL showing enhanced important for written words and speech output, and the right ATL more strongly linked with visual face stimuli (Rice, Hoffman, & Lambon Ralph, 2015; Rice, Lambon Ralph, & Hoffman, 2015).

#### 4.1.2 The lateral frontal cortex

It is a packed region that serves multiple functions – motor planning, phonological processing and executive control (Fedorenko et al., 2013; Hickok, 2012; Indefrey & Levelt, 2004; Lambon Ralph et al., 2017). While major neurocognitive models highlight the importance of left frontal areas in articulatory motor control (Hickok, 2012; Hickok et al., 2011), in the current study both tasks also activated frontal areas that are associated with semantic control and domain-general executive processes (Fedorenko et al., 2013; Jackson, 2021). While the executive demands of category fluency are clear (as discussed in detail below), executive control may also be important for picture naming in order to resolve which legitimate word to use for each concept (e.g., the hyponym problem: (Levelt, 1992)) as well as competition between the target vs. phonologically- and semantically-related words (de Zubicaray et al., 2001, 2006). Therefore, the engagement of executive control mechanisms could be regarded as a core component of the speech production network.

#### 4.1.3 The anterior temporal lobe

The current data also highlight the importance of the ATL as a core-component of the speech production system. Reliable ATL activation was absent in our meta-analysis of the existing literature, presumably due to the poor detection of the signal in this region when using conventional (single echo) fMRI. Indeed, as per other examinations of ATL semantic function (Lambon Ralph et al., 2017), when using fMRI acquisition with improved signal detection then strong ATL activation was found across tasks (Fig. 2). Accordingly, this finding helps to realign the neuroimaging literature with evidence from patient studies, whereby damage to the ATL is associated with severe anomia, as well as a generalized semantic impairment across receptive and expressive domains and multiple modalities. Together, these results are consistent with the hypothesis that the ATL acts a representational hub for transmodal and transtemporal semantic representation (Jackson et al., 2021; Lambon Ralph et al., 2017). Indeed, convergent evidence to support the semantic hub hypothesis is found across methodological techniques. For instance, when using cortical grid-electrodes, stimulation of the ATL in patients is associated with naming errors (Rogers et al., 2021; Shimotake et al., 2014) and electrocorticography (ECoG) data have shown ATL semantic coding using multivariate methods (Rogers et al., 2021). Additionally, transcranial magnetic stimulation (TMS) of the ATL impairs responses in naming and comprehension (Pobric et al., 2010; Woollams et al., 2017).

Although both fluency and naming tasks engaged the ATL, the ATL were more strongly engaged by picture naming. Perhaps this is not surprising given that the ATL would be engaged by at least two form of semantic retrieval in the picture naming task—first by processes associated with visual object recognition, and then by processes associated with name retrieval. Indeed, during picture naming there are numerous and extended top-down and bottom-up interaction between the ATL and visual areas, and this elongated process presumably leads to boosted ATL activation (Chan et al., 2011). Actually, the fact that we find ATL activation for verbal fluency at all could be considered surprising, given the ATLs’ anatomical location at the most anterior section of the ventral visual stream, and hence the common suggestion that this region is primarily involved in visual recognition (Duchaine & Yovel, 2015; Kravitz et al., 2011; Skipper et al., 2011).

### 4.2 Dorsal vs. ventral language pathways

In the Introduction, we noted that Geschwind’s classic language model centered speech production on the dorsal language pathway (with these notions retained in many contemporary dual language pathway theories: (Hagoort, 2005; Hickok, 2012; Hickok & Poeppel, 2007; Hickok et al., 2011; Indefrey & Levelt, 2004)), yet recent studies have indicated that Wernicke not only contemplated a dual-pathway architecture but considered the dorsal pathway to be minor (capable of mimicking) and the ventral pathway to be the dominant (Weiller et al., 2011). Thus, notions fit with one of the only implemented neurocomputational models of the dual language pathways (Ueno et al., 2011) which demonstrated an emergent division of labor such that the dorsal pathway specializes in extracting phonological structures and thus is key to repetition (and generalization to nonword repetition) and the ventral for mappings into and out of meaning. Indeed, this framework is thus able to simulate the double dissociations between conduction aphasia (impaired repetition but preserved comprehension after temporo-parietal damage) and semantic dementia (degrading concepts but preserved repetition in the context of atrophy centered on the ATLs).

Turning to the results from this study, the posterior temporal and inferior parietal regions are notable by their absence in semantically-driven speech production. This was true when considering the functional neuroimaging as a whole through the ALE analysis, as well as in the targeted fMRI study. Even targeted ROIs in the SMG and core AG failed to reveal positive engagement with the tasks (if anything being de-activated relative to the control task). It should, perhaps, be noted that such ventral IPL regions are observed in more extended speech production tasks, though another recent combined ALE and targeted fMRI exploration (Humphreys et al., 2024) found (a) a similar network of frontal and anterior temporal regions engaged as the present study; while (b) the AG was only positively engaged when participants were required to retrieve information from their autobiographical memory but not in equally elaborate semantic definitions (when it, again, deactivated). In contrast, the speech production network itself was positively engaged irrespective of the speech topic.

#### 4.2.1 The AG

Prominent models propose that the AG functions as a semantic hub that stores semantic information (Binder & Desai, 2011; Binder et al., 2009; Geschwind, 1965). In the current study of semantically-driven speech tasks, we found no evidence to support this suggestion in either the meta-analysis or fMRI study. Indeed, unlike the ATL and the frontal regions, the AG was systematically deactivated by all tasks relative to rest, with greater or equal levels of deactivation for the semantic tasks relative to the control condition. This is consistent with other speech production studies where the AG is not reliably activated by semantic speech production tasks (Humphreys et al., 2024), and where the AG shows greater deactivation for speech production relative to performing non-meaningful tongue movements (Geranmayeh et al., 2012). Parietal damage does not result in gross semantic memory retrieval impairment, unlike damage to the ATL (Jefferies & Lambon Ralph, 2006). The current data add to a growing body of evidence that the AG might not actively contribute to all forms of semantic processing. For instance, direct contrasts have shown that while the ATL is positively engaged by semantic tasks, the AG is equally deactivated for semantic and non-semantic control tasks (consistent with AGs role as part of the default mode network (DMN) (Buckner et al., 2008)). Furthermore, when processing matched semantic and non-semantic tasks, the AG shows a main effect of task difficulty (easy > hard) but no semantic vs. non-semantic difference (Humphreys & Lambon Ralph, 2017; Quillen et al., 2021) and compellingly, classic examples of apparent semantic effects (word > non-word, concrete > abstract) can be flipped by reversing the difficulty of the task or the stimuli (Graves et al., 2017; Pexman et al., 2007). Finally, while the AG is deactivated by many cognitive domains, including semantic, there are certain tasks—perhaps most prominently those involving episodic memory—that do positively engage this region (Gonzalez et al., 2015; Humphreys et al., 2024; Humphreys et al., 2020; Humphreys & Lambon Ralph, 2015; Sestieri et al., 2017). Together, these results suggest that the AG is not a core component of the speech production network, and instead serves alternative functions (Buckner & Carroll, 2007; Humphreys & Lambon Ralph, 2015; Humphreys et al., 2021; Humphreys & Tibon, 2022; Wagner et al., 2005).

### 4.3 Task-specific variance in the extent of lateralisation

Despite an overall bilateral, asymmetric speech production network, the extent of lateralization was found to be task dependent, with fluency showing boosted left hemisphere engagement compared to naming, particularly in frontal areas. A simple comparison of standard (multi-word) fluency vs. picture (single) naming might suggest that the extent of left-hemisphere bias is dependent on the extent to which a task involved rapid and fluid speech production and complex articulatory coordination. Such differences are unlikely to explain the current results since the two tasks were matched for speech rate and the number of words produced. Furthermore, the result is consistent with existing findings where rapid and fluent speech does not appear to be the driving force behind task-specific variations in laterality (Blank et al., 2002; Geranmayeh et al., 2012).

Instead, the locus of activation overlapped with regions associated with semantic and domain-general control. Category fluency loads heavily onto executive tasks (Schumacher et al., 2022) and patients with semantic control impairments show much greater category fluency deficits compared to naming (Noonan et al., 2010; Robinson et al., 1998). Furthermore, category fluency correlates highly with overall disease severity in in patients with Alzheimer’s disease (Lambon Ralph et al., 2003) and is poor at differentiating between patient groups despite clear and considerable variation in their language abilities (Henderson et al., 2024). This is consistent with the notion that category fluency is a multi-faceted task, relying not only on the speech production system but also regions associated with semantic control, working memory, inhibitory control etc. in the verbal and non-verbal domain (Henderson et al., 2024; Lambon Ralph et al., 2003; Schumacher et al., 2022). Thus, one possible reason for the enhanced leftward lateralisation of fluency over naming tasks is that, since the semantic control network is left-lateralized (Jackson, 2021), when a semantic task requires more executive resources the network is increasingly shifted leftwards due to interactions with the semantic control system. Nevertheless, the function of hemispheric specialization, and moreover what is the driving force behind such functional biases remains an open question and a considerable amount of research is needed to understand this puzzling issue.

### 4.4 Clinical implications

As set out in detail in the Introduction, the current findings are also of clinical relevance: 1) In terms of neuropsychological language assessment, while category fluency may be considered a quick and easy speech measure, it is far from a pure measure of speech production as failure can occur even without language impairment given into increased executive demands (Noonan et al., 2010; Robinson et al., 1998). Indeed, caution should be drawn against the over-reliance on any single assessment of language function given the task-dependent nature of performance and thus more interpretable neuropsychological patterns emerge when contrastive tasks are administered in parallel (indeed, inspiring the logic of the present study). 2) In terms of use in neurosurgical settings, it is critically important to take into account task-specific variations in functional engagement. For instance, an over-reliance on picture-naming alone could downplay the functional relevance of executive functions that are, nevertheless, critically important for language function. Or alternatively, and overreliance on category fluency might downplay the role of the right-hemisphere and/or the object recognition in meaningful speech production (Birn et al., 2010; Blank et al., 2002; Geranmayeh et al., 2012; Moore & Price, 1999; Vitali et al., 2005; Zelkowicz et al., 1998). 3) More generally, if using fMRI to explore language functions in patient populations then the task and fMRI acquisition choices are very important for the ability to see the full speech production neural network (Rice et al., 2018; Robson et al., 2014).

## 5 Conclusions

The current study combined the results of a meta-analysis and fMRI study, directly contrasting picture-naming and category fluency tasks. The results suggest that the core speech production network comprises a bilateral fronto-temporal network including executive and motor frontal areas as well as ATL semantic representational regions. The extent of lateralization in frontal regions for speech production is task-dependent with more executively-demanding speech tasks (in this case category fluency) associated with boosted left hemisphere activation.

## Open Access

For the purpose of open access, the UKRI-funded authors have applied a Creative Commons Attribution (CC BY) licence to any Author Accepted Manuscript version arising from this submission.

## Supplementary Material

Supplementary Material

## Data Availability

The data are available at MRC’s central open science repository: http://www.mrc-cbu.cam.ac.uk/publications/opendata and can be downloaded from OSF at https://osf.io/pe7un/
