# Supplementary material for: Mapping the task-general and task-specific neural correlates of speech production: Meta-analysis and fMRI direct comparisons of category fluency and picture naming
Source: Imaging Neurosci (Camb). 2025 Sep 26;3:IMAG.a.154. doi: 10.1162/IMAG.a.154 (PMC12477641; doi:10.1162/IMAG.a.154)

Supplementary

Table S1. The clusters identified from the meta-analysis of Category Fluency and Picture Naming studies

	Cluster #	x	y	z	Z	Anatomical label
Fluency studies	1	-44	24	24	8.489372	Left Middle frontal gyrus
	1	-42	8	26	6.206844	Left Inferior frontal gyrus
	1	-46	-12	36	5.598102	Left Precentral gyrus
	2	-6	12	44	10.21135	Left Supplementary Motor Area
	3	-24	-2	50	6.453395	Left Middle frontal gyrus
	4	-14	-8	16	5.2552	Left Thalamus
	5	-28	-58	32	3.411601	Left Middle Temporal Gyrus
	6	48	-10	38	4.959919	Right Precentral gyrus
	7	-36	-36	-2	4.334408	Left Caudate
	8	42	10	32	3.678642	Right Precentral gyrus
	9	40	26	-10	3.606574	Right Inferior frontal gyrus
	10	36	-62	-28	3.189645	Right Cerebellum
	11	40	36	16	3.146989	Right Middle Frontal Gyrus
	12	-2	2	68	3.204904	Left Superior Frontal Gyrus
Naming studies	1	-44	-60	-16	7.750345	Left Fusiform Gyrus
	1	-36	-44	-16	7.008279	Left Fusiform Gyrus
	1	-38	-82	-8	4.462598	Left Fusiform Gyrus
	2	-48	10	8	6.025524	Left Precentral gyrus
	2	-46	28	2	5.835731	Left Inferior frontal gyrus
	2	-44	8	28	5.772944	Left Inferior frontal gyrus
	2	-48	18	24	5.729263	Left Middle frontal gyrus
	2	-34	24	2	4.468136	Left Insula
	3	32	-48	-14	4.616426	Right Cerebellum
	4	-6	8	54	4.353867	Left Supplementary Motor Area
	5	34	-88	10	4.809121	Right Occipital lobe
	6	-52	-4	48	5.190566	Left Precentral gyrus
	7	-28	-96	8	4.692374	Left Occipital Lobe
	8	-54	-30	0	3.677164	Left Middle Temporal Gyrus
	9	58	-4	38	3.611051	Right Precentral gyrus

Table S2. The peak coordinates of activation from the fMRI study.

Uncorrected p < .001, cluster corrected					
Contrast	t	x	y	z	Anatomical label
Fluency > control	18.03	-3	14	48	Cingulate gyrus/motor cortex
	15.36	-5	20	38	Paracingulate gyrus
	15.18	13	-77	-29	Cerebellum
	14.14	-30	27	-3	Orbito-frontal cortex
	13.71	20	25	31	Cingulate gyrus
	13.41	-30	22	3	Insular
	12.86	-39	-3	51	Precentral gyrus
	12.11	-42	21	25	inferior frontal gyrus, BA44
	11.67	12	16	37	Cingulate gyrus
	10.22	-26	-74	35	Superior parietal lobule

	10.1	36	18	3	Insular
	9.41	36	21	-13	Orbito-frontal cortex
	8.82	16	4	19	Caudate
	8.37	-9	-72	7	Visual cortex
	7.86	-4	-8	6	Thalamus
	7.83	-14	-3	14	Caudate
	7.76	-30	60	6	Frontal pole
	7.13	52	-12	41	Postcentral gyrus
	6	-36	-25	-26	Fusiform gyrus
	5.47	38	-21	-25	Fusiform gyrus
Naming > control	17.28	36	-61	-16	Fusiform gyrus
	16.96	46	-71	17	Lateral occipital cortex
	16.67	-2	8	48	Cingulate gyrus/motor cortex
	15.4	34	-47	-21	Fusiform gyrus
	14.54	-35	-85	-9	Lateral occipital cortex
	14.24	-33	-69	-18	Fusiform gyrus
	13.96	-36	-73	-18	Fusiform gyrus
	12.16	-28	-74	26	Lateral occipital cortex
	11.56	-29	26	1	Insular
	10.62	54	-11	42	Postcentral gyrus
	10.11	-43	-13	42	Middle frontal gyrus
	9.95	40	-21	-25	Fusiform gyrus
	8.3	-39	20	25	inferior frontal gyrus, BA44
	8.3	-31	-58	55	Superior parietal lobule
	7.57	-36	-27	-25	Fusiform gyrus
	7.25	-47	6	38	inferior frontal gyrus, BA44
Fluency > naming	11.48	-6	15	48	Cingulate gyrus/premotor cortex
	10.75	-27	12	48	Middle frontal gyrus
	9.85	-3	24	36	Cingulate gyrus
	9.04	15	-87	-39	Cerebellum
	8.97	12	27	30	Cingulate gyrus
	8.71	-24	54	0	Frontal pole
	8.63	-45	27	24	Middle frontal gyrus
	8.29	12	-81	-30	Cerebellum
	7.74	33	36	30	Frontal pole
	7.52	-3	12	63	Superior frontal gyrus
	7.32	27	9	57	Middle frontal gyrus
	7.04	-9	33	18	Anterior cingulate cortex
	6.86	0	30	15	Anterior cingulate cortex
	6.56	9	36	15	Anterior cingulate cortex
	6.52	-36	-78	42	Angular gyrus, PGp
	5.92	-24	33	30	Middle frontal gyrus
	5.342	21	3	69	Premotor cortex
	5.34	-15	63	15	Frontal pole

	5.17	-3	39	51	Superior frontal gyrus
	4.60	-48	30	3	Inferior frontal gyrus, BA45
Naming > fluency	22.43	36	-57	-15	Fusiform gyrus
	21.82	39	-69	-12	Lateral occipital cortex
	19.72	-36	-84	-6	Lateral occipital cortex
	18.81	-39	-78	-12	Lateral occipital cortex
	18.77	-33	-90	12	Occipital pole
	17.93	36	-84	6	Lateral occipital cortex
	16.47	33	-84	15	Fusiform gyrus
	15.45	30	-72	30	Lateral occipital cortex
	10.2944	30	-57	57	Superior parietal lobule
	10.15	30	-54	51	Superior parietal lobule/Intra-parietal sulcus
	8.15	42	6	27	Precentral gyrus
	7.67	51	33	9	Inferior frontal gyrus, BA45
	6.39	-36	-6	15	Insular
	6.06	-66	-27	30	Supramarginal gyrus
	6.05	-66	-15	30	Postcentral gyrus
	5.95	24	-3	-12	Amygdala
	5.93	66	-21	36	Supramarginal gyrus
	5.76	36	-9	12	Insular
	5.69	24	0	-6	Putamen
	5.57	33	-9	-42	Fusiform gyrus
	5.54	-24	-3	-15	Amygdala
	5.49	-57	0	33	Precentral gyrus
	5.36	18	-30	-3	Thalamus
	5.31	-30	-54	51	Superior parietal lobule
	5.24	-24	-63	48	Superior parietal lobule
	5.14	-27	-3	-3	Putamen
	5.00	27	3	3	Putamen
	4.61	-51	-30	57	Postcentral gyrus

Figure S1. The results from the fMRI study for the contrasts Fluency > Rest (red) and Naming > Rest (green) (thresholded at $p < .001$, cluster corrected using FWE $p < .05$). The overlap between each network is shown in yellow.

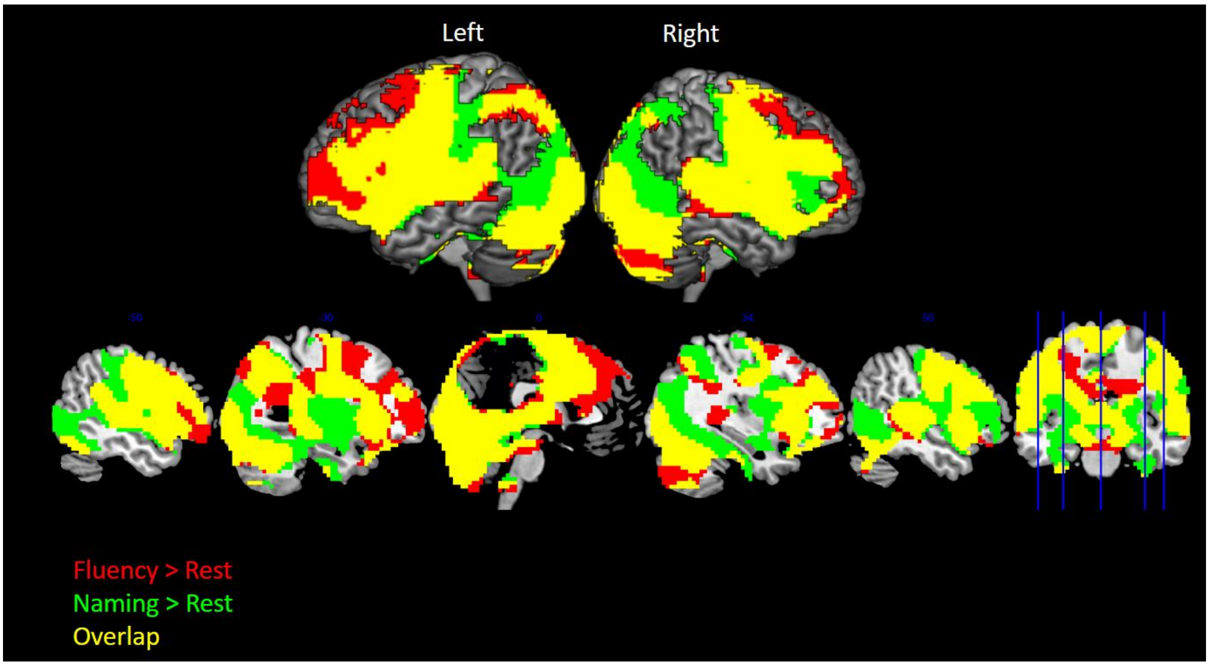

Supplement: Supplementary Material [file IMAG.a.154_supp.pdf]
